# Supplementary material for: Structural organization of a major neuronal G protein regulator, the RGS7-Gβ5-R7BP complex
Source: eLife. 2018 Dec 12;7:e42150. doi: 10.7554/eLife.42150 (PMC6310461; doi:10.7554/eLife.42150)
Supplement: Figure 5—source data 1. [file elife-42150-fig5-data1.docx]

**Figure 5-source data 1. RGS7-Gβ5 communities**

| Community | Residues |
| --- | --- |
| 1 | RGS7: 19 20 21 22 23 24 25 26 27 28 29 30 31 32 33 34 35 36 37 38 39 40 41 42 43 44 45 46 48 49 50 51 52 53 54 55 56 57 58 59 60 61 62 63 64 65 66 67 68 69 70 71 72 73 74 75 76 77 78 79 80 81 82 83 84 85 86 87 88 89 90 91 92 93 94 95 96 97 98 99 100 101 102 103 104 105 106 107 108 109 110 111 112 113 114 115 116 117 118 119 120 121  Gβ5: 280 281 283 284 304 322 |
| 2 | RGS7: 47  Gβ5: none |
| 3 | RGS7: 225  Gβ5: none |
| 4 | RGS7: 396  Gβ5: none |
| 5 | RGS7: none  Gβ5: 4 |
| 6 | RGS7: none  Gβ5: 104 |
| 7 | RGS7: 213 216 218 219 220 268 270 271 272 273 274  Gβ5: 33 192 193 194 196 197 198 199 200 202 204 206 207 208 209 210 211 213 214 215 216 217 218 219 220 221 222 223 224 230 231 232 233 234 235 236 237 238 239 240 241 242 243 244 245 247 251 253 254 256 257 258 259 260 261 262 264 265 266 271 273 275 276 278 285 303 |
| 8 | RGS7: 317 319 320 321 322 323 324 325 326 327 328 329 330 331 332 333 334 335 336 337 338 339 340 341 342 343 344 345 346 347 348 349 350 351 352 353 354 355 357 360 430 432 433 434 435 436 437 438 439 440 441 442 443 444 445 446 447 448 449 450  Gβ5: none |
| 9 | RGS7: 227 229 231 232 233 234 235 236 237 238 239 240 241 242 243 244 245 246 247 248 249 250 251 252  Gβ5: none |
| 10 | RGS7: 214 215 217  Gβ5: 105 107 109 112 114 115 116 121 123 124 125 126 127 128 129 148 149 150 151 152 153 154 155 156 157 158 159 160 161 162 164 165 168 169 170 171 172 173 174 175 176 177 178 179 180 181 182 183 184 185 186 187 189 190 191 201 203 212 225 226 228 229 |
| 11 | RGS7: 122 123 124 125 126 127 128 129 130 131 132 133 134 135 136 137 138 139 140 141 142 143 144 145 146 147 148 149 150 151 152 153 154 155 156 157 158 159 160 161 162 163 164 165 166 167 168 169 170 171 172 173 174 175 176 177 178 179 180 181 182 183 184 185 186 187 188 189 190 191 192 193 194 195 196 197 198 199 200 201 202 203 204 206  Gβ5: none |
| 12 | RGS7: 253 254 255 256 257 258 259 260 261 262 263 264 265 266 267 269  Gβ5: 5 6 7 8 9 10 11 12 13 14 15 16 17 18 19 20 21 22 23 24 25 26 27 28 29 30 31 32 34 |
| 13 | RGS7: 295 299 300 301 302 303 304 305 306 307 308 309 310 311 312 313 314 315 316 318  Gβ5: 50 52 54 55 56 57 71 73 75 93 333 334 335 336 337 338 339 340 351 352 353 |
| 14 | RGS7: 221 222 223 224 226 228 230  Gβ5: 195 |
| 15 | RGS7: 356 358 359 361 362 363 364 365 366 367 368 369 370 371 372 373 374 375 376 377 378 379 380 381 382 383 384 385 386 387 388 389 390 391 392 393 394 395 397 398 399 400 401 402 403 404 405 406 407 408 409 410 411 412 413 414 415 416 417 418 419 420 421 422 423 424 425 426 427 428 429 431  Gβ5: 163 166 167 188 227 |
| 16 | RGS7: 275 276 277 278 279 280 281 282 283 284 285 286 287 288 289 290 291 292 293 294 296 297 298  Gβ5: 35 36 37 38 39 40 41 42 43 44 45 46 47 48 49 51 53 205 246 248 249 250 252 255 263 267 268 269 270 272 274 277 279 282 286 287 288 289 290 291 292 293 294 295 296 297 298 299 300 301 302 306 307 308 309 310 311 312 313 314 315 316 317 318 319 320 321 328 331 |
| 17 | RGS7: 205 207 208 209 210 211 212  Gβ5: none |
| 18 | RGS7: none  Gβ5: 58 59 60 61 62 63 64 65 66 67 68 69 70 72 74 76 77 78 79 80 81 82 83 84 85 86 87 88 89 90 91 92 94 95 96 97 98 99 100 101 102 103 106 108 110 111 113 117 118 119 120 122 130 131 132 133 134 135 136 137 138 139 140 141 142 143 144 145 146 147 305 323 324 325 326 327 329 330 332 341 342 343 344 345 346 347 348 349 350 |
