# Supplementary material for: Structural organization of a major neuronal G protein regulator, the RGS7-Gβ5-R7BP complex
Source: eLife. 2018 Dec 12;7:e42150. doi: 10.7554/eLife.42150 (PMC6310461; doi:10.7554/eLife.42150)
Supplement: Figure 5—source data 2. [file elife-42150-fig5-data2.docx]

**Figure 5-source data 2. RGS7-Gβ5 critical nodes**

| Communities linked by the critical nodes | Residue X | Residue Y | Betweenness score |
| --- | --- | --- | --- |
| 1-7 | RGS7:22 | Gβ5:240 | 61202.542969 |
| 1-11 | RGS7:121 | RGS7:123 | 42164.183594 |
| 1-16 | Gβ5:284 | Gβ5:302 | 28684.332031 |
| 1-16 | RGS7:77 | Gβ5:320 | 23518.222656 |
| 1-17 | RGS7:88 | RGS7:209 | 73.750000 |
| 1-18 | Gβ5:322 | Gβ5:324 | 1735.500000 |
| 7-9 | RGS7:219 | RGS7:236 | 31432.000000 |
| 7-10 | Gβ5:157 | Gβ5:199 | 27852.294922 |
| 7-10 | Gβ5:171 | Gβ5:198 | 36428.421875 |
| 7-12 | RGS7:267 | RGS7:271 | 22648.664062 |
| 7-12 | RGS7:266 | RGS7:270 | 24361.691406 |
| 7-14 | Gβ5:195 | Gβ5:218 | 7000.000000 |
| 7-16 | Gβ5:266 | Gβ5:274 | 35896.121094 |
| 7-16 | RGS7:274 | RGS7:276 | 39031.191406 |
| 7-18 | Gβ5:303 | Gβ5:327 | 1305.104248 |
| 8-13 | RGS7:315 | RGS7:319 | 15437.293945 |
| 8-15 | RGS7:427 | RGS7:430 | 14128.538086 |
| 8-16 | RGS7:293 | RGS7:329 | 1440.374756 |
| 9-12 | RGS7:251 | RGS7:253 | 6317.000000 |
| 9-14 | RGS7:230 | RGS7:232 | 3432.000000 |
| 10-13 | Gβ5:73 | Gβ5:115 | 8.000000 |
| 10-15 | Gβ5:167 | Gβ5:180 | 98400.968750 |
| 10-16 | Gβ5:203 | Gβ5:205 | 1318.333252 |
| 10-17 | RGS7:212 | RGS7:214 | 25916.865234 |
| 10-18 | Gβ5:68 | Gβ5:109 | 23642.775391 |
| 11-17 | RGS7:203 | RGS7:205 | 19438.951172 |
| 13-16 | Gβ5:291 | Gβ5:333 | 6610.305176 |
| 13-16 | Gβ5:307 | Gβ5:352 | 5966.556152 |
| 13-18 | Gβ5:91 | Gβ5:93 | 11819.332031 |
| 13-18 | Gβ5:57 | Gβ5:350 | 10263.083984 |
| 16-18 | Gβ5:328 | Gβ5:344 | 34741.566406 |
