# Supplementary material for: Structural organization of a major neuronal G protein regulator, the RGS7-Gβ5-R7BP complex
Source: eLife. 2018 Dec 12;7:e42150. doi: 10.7554/eLife.42150 (PMC6310461; doi:10.7554/eLife.42150)
Supplement: Figure 5—source data 3. [file elife-42150-fig5-data3.docx]

**Figure 5-source data 3. RGS9-Gβ5 communities**

| Community | Residues |
| --- | --- |
| 1 | RGS9: 7 8 9 10 11 12 112 203 204 205 206 207 208 209 210 211 212  Gβ5: 155 173 197 199 |
| 2 | RGS9: none  Gβ5: 50 52 |
| 3 | RGS9: 14 15 16 17 18 19 20 21 22 23 24 25 26 27 28 29 30 31 32 33 34 35 36 37 38 39 40 42 47 48 49 50 51 52 53 54 55 56 57 58 59 60 61 62 63 64 65 66 67 68 70 71 73 74 75 77 78 79 80 81 82 83 84 85 86 87 88 89 90 91 92 93 94 95 96 97 98 99 100 101 102 103 104 105 106 107 108 109 110 111 113 198 200  Gβ5: 280 281 283 |
| 4 | RGS9: 282 283 284 285 286 287 288 289 290 291 292 293 294 295 296 297 298 299 300 301 302 303 304 305 306 307 308 309 310 311 312 313 314 315 316 317 318 319 320 321 322 323 324 325 326 327 328 329 330 331 332 333 334 336 362 363 364 365 388 389 390 391 392 393 394 395 396 397 398 399 400 401 402 403 404 405 406 407 408 409 410 411 412 413 414 415 416 417 418 419 421  Gβ5: 163 206 207 208 209 |
| 5 | RGS9: 213 214 215 216 217 218 219 220 221 222 223 224 225 226 227 228 229 230 231 232 233 234 235 236 237  Gβ5: 9 10 11 12 13 14 15 16 17 18 19 20 21 22 23 24 25 26 27 28 29 30 31 32 33 35 271 |
| 6 | RGS9: 13  Gβ5: 192 193 194 195 196 200 202 204 213 214 215 216 217 218 219 220 221 222 223 224 230 231 232 233 234 235 236 237 238 239 240 241 242 245 247 251 253 254 256 259 260 262 264 266 268 273 275 276 278 |
| 7 | RGS9: 41 43 44 45 46 114 115 116 117 118 119 120 121 122 123 124 125 126 127 128 129 130 131 132 133 134 135 136 137 138 139 140 141 142 143 144 145 146 147 148 149 150 151 152 153 154 155 156 157 158 159 160 161 162 163 164 165 166 167 168 169 170 171 172 173 174 175 176 177 178 179 180 181 182 183 184 185 186 187 188 189 190 191 192 193 194 195 196 197 199 201  Gβ5: none |
| 8 | RGS9: 259 263 264 265 266 267 268 269 270 271 272 273 274 275 276 277 278 279 280 281 420  Gβ5: 54 56 57 58 59 69 71 76 92 93 95 332 333 334 335 336 337 338 339 341 349 351 353 |
| 9 | RGS9: 335 338 339 340 341 342 343 344 345 346 347 348 349 350 351 352 353 354 355 356 357 358 359 360 361 366 367 368 369 370 371 372 373 374 375 376 377 378 379 380 381 382 384 385 387  Gβ5: none |
| 10 | RGS9: 69 72 76 202 260  Gβ5: 51 53 55 66 243 244 255 257 258 261 263 265 277 279 282 284 285 286 287 288 289 290 298 299 300 301 302 303 304 305 306 307 308 309 310 311 315 316 317 318 319 320 321 322 323 324 325 326 327 328 329 330 331 340 342 343 344 345 346 348 350 352 |
| 11 | RGS9: 337 383 386  Gβ5: 68 73 109 110 115 123 124 126 127 128 129 148 149 150 151 152 153 154 156 157 158 160 162 164 165 166 167 168 169 170 171 172 174 175 176 177 178 179 180 181 182 183 184 185 186 187 188 189 190 191 198 201 203 210 211 212 225 226 227 228 229 |
| 12 | RGS9: 238 239 240 241 242 243 244 245 246 247 248 249 250 251 252 253 254 255 256 257 258 261 262  Gβ5: 34 36 37 38 39 40 41 42 43 44 45 46 47 48 49 205 246 248 249 250 252 267 269 270 272 274 291 292 293 294 295 296 297 312 313 314 |
| 13 | RGS9: none  Gβ5: 60 61 62 63 64 65 67 70 72 74 75 77 78 79 80 81 82 83 84 85 86 87 88 89 90 91 94 96 97 98 99 100 101 102 103 104 105 106 107 108 111 112 113 114 116 117 118 119 120 121 122 125 130 131 132 133 134 135 136 137 138 139 140 141 142 143 144 145 146 147 159 161 347 |
