# Supplementary material for: Structural organization of a major neuronal G protein regulator, the RGS7-Gβ5-R7BP complex
Source: eLife. 2018 Dec 12;7:e42150. doi: 10.7554/eLife.42150 (PMC6310461; doi:10.7554/eLife.42150)
Supplement: Figure 5—source data 4. [file elife-42150-fig5-data4.docx]

**Figure 5-source data 4. RGS9-Gβ5 critical nodes**

| Communities linked by the critical nodes | Residue X | Residue Y | Betweenness score |
| --- | --- | --- | --- |
| 1-3 | RGS9:12 | RGS9:14 | 22022.082031 |
| 1-5 | RGS9:210 | RGS9:213 | 11875.041992 |
| 1-6 | RGS9:207 | Gβ5:241 | 5285.582031 |
| 1-7 | RGS9:201 | RGS9:203 | 2456.750000 |
| 1-10 | RGS9:202 | RGS9:204 | 6114.000000 |
| 1-11 | Gβ5:127 | Gβ5:155 | 9972.833984 |
| 1-13 | Gβ5:125 | Gβ5:155 | 3156.083008 |
| 3-6 | RGS9:19 | Gβ5:260 | 20270.376953 |
| 3-7 | RGS9:79 | RGS9:190 | 69890.835938 |
| 3-10 | RGS9:76 | RGS9:79 | 73084.500000 |
| 4-6 | Gβ5:204 | Gβ5:206 | 5093.750000 |
| 4-8 | RGS9:280 | RGS9:418 | 22674.500000 |
| 4-9 | RGS9:385 | RGS9:388 | 12384.917969 |
| 4-11 | RGS9:336 | Gβ5:167 | 20330.417969 |
| 4-12 | RGS9:254 | RGS9:295 | 48112.332031 |
| 5-6 | RGS9:228 | Gβ5:233 | 22952.664062 |
| 5-12 | Gβ5:31 | Gβ5:34 | 15161.206055 |
| 6-10 | Gβ5:200 | Gβ5:243 | 34929.878906 |
| 6-11 | Gβ5:224 | Gβ5:226 | 35783.585938 |
| 6-12 | Gβ5:253 | Gβ5:267 | 8836.874023 |
| 7-10 | RGS9:199 | RGS9:202 | 6661.000000 |
| 8-10 | Gβ5:57 | Gβ5:350 | 18863.333984 |
| 8-11 | Gβ5:71 | Gβ5:73 | 1397.750000 |
| 8-12 | Gβ5:246 | Gβ5:332 | 6997.166504 |
| 8-13 | Gβ5:91 | Gβ5:93 | 13269.416016 |
| 9-11 | RGS9:383 | RGS9:385 | 37777.917969 |
| 10-11 | Gβ5:66 | Gβ5:68 | 8102.333984 |
| 10-12 | Gβ5:296 | Gβ5:310 | 31774.957031 |
| 10-13 | Gβ5:66 | Gβ5:81 | 23107.582031 |
| 11-12 | Gβ5:203 | Gβ5:205 | 649.000000 |
| 11-13 | Gβ5:131 | Gβ5:182 | 10609.333008 |
| 11-13 | Gβ5:161 | Gβ5:167 | 8356.250000 |
